# Microbiota Analysis of Ejaculated Honey Bee Drone Semen and the Effect of Semen Collection Method on Bacterial Loads

**DOI:** 10.3390/insects15060377

**Published:** 2024-05-22

**Authors:** Jesús Yániz, Marion Toquet, Pilar Santolaria, Miguel Angel Silvestre, Raquel Toledo-Perona, Ángel Gómez-Martín

**Affiliations:** 1 BIOFITER Research Group, Environmental Sciences Institute (IUCA), Department of Animal Production and Food Sciences, University of Zaragoza, 22071 Huesca, Spain; psantola@unizar.es; 2 Microbiological Agents Associated with Animal Reproduction (ProVaginBIO) Research Group, Departamento Producción y Sanidad Animal, Salud Pública Veterinaria y Ciencia y Tecnología de los Alimentos, Facultad de Veterinaria, Universidad Cardenal Herrera-CEU, CEU Universities, Carrer Tirant lo Blanc, 7, Alfara del Patriarca, 46115 Valencia, Spain; marion.toquet1@uchceu.es (M.T.); raquel.toledoperona@uchceu.es (R.T.-P.); angel.gomezmartin@uchceu.es (Á.G.-M.); 3 Department of Cellular Biology, Functional Biology and Physical Anthropology, University of Valencia, 46100 Burjassot, Spain; miguel.silvestre@uv.es

**Keywords:** *Apis mellifera*, sperm quality, bacteriospermia, microbiome, instrumental insemination

## Abstract

**Simple Summary:**

A bacterial presence in semen may reduce sperm viability and increase the risk of infection transmission to the queen after artificial insemination in honey bees. The aims of this study were to characterize and compare the microbiota of honey bee drone semen from different locations and to determine the effect of semen collection method on bacterial loads. The results of the microbial composition analyses were described, showing differences between apiaries and colonies in the composition and abundance of the seminal microbiota. The collection method had a great impact on the degree of the bacterial loads of semen samples, with the traditional ejaculation method more favorable than the collection of semen from the seminal vesicles.

**Abstract:**

Artificial insemination in queen honey bees is the only tool that provides complete control over mating for research and breeding purposes, making it essential in genetic improvement and conservation programs in this species. The aims of this study were to characterize drone semen bacterial loads by culture-dependent and independent methods and to describe their variation depending on the method of semen collection, the colony and the apiary. In the first experiment, the bacterial loads of semen collected from the seminal vesicles or from ejaculates was studied using culture-dependent methods. The collection method had a significant influence on the overall bacterial count in semen. Out of the 42 semen samples analyzed, 26 (61.9%) tested positive for bacterial isolation. This encompassed the entirety of samples obtained from the seminal vesicles (21 of 21), whereas only 23.8% of those derived from ejaculates (5 out of 21) showed bacterial isolation. In the second experiment, next-generation sequencing techniques were used to describe the microbiome of ejaculated drone semen for the first time. The most abundant phyla were Proteobacteria, Firmicutes, Bacteroidota and Actinobacteriota, while the most abundant genera were *Lactobacillus, Staphylococcus*, *Prevotella*, *Alloprevotella* and *Streptococcus*. The results showed that the apiary had a significant effect on the community structure composition and abundance of the seminal microbiota, and significative differences in abundance were observed for the genera *Sphingomonas*, *Methylobacterium-Methylorubrum*, *Bifidobacterium* and *Alloprevotella*. Significant differences were also observed in the richness of the microbiota between apiaries and colonies.

## 1. Introduction

Artificial insemination (AI) in queen honey bees (*Apis mellifera*) is the only tool that provides complete control over mating for research and breeding purposes, making it essential in genetic improvement and conservation programs in this species [1]. Furthermore, when combined with the creation of semen banks, AI allows for the recovery of valuable genetics that may have been lost due to various reasons, such as queen mortality, contamination from other subspecies of bees, or when there is a need to reverse undesirable effects of a genetic improvement program. It also enables the production of mated queens in cases when unfavorable weather conditions or the presence of predators hinder natural mating.

The quality of semen is a key component of artificial insemination [2]. Semen can be contaminated with bacteria during collection or further processing, even when performed under careful conditions. This contamination reduces sperm viability and increases the risk of infection transmission to the female after AI [3,4]. Bacterial contamination is also a risk to the establishment of semen banks. Nonetheless, there exists certain evidence suggesting that lactobacilli, one of the bacteria present in semen, may have a positive impact on sperm quality [5]. Despite the assumption that a low microbial contamination of semen is a fundamental element to its preservation and the outcomes of insemination [4], bacterial contamination is fairly well studied. Using conventional plate culture techniques, Andere et al. [6] analyzed the bacteria of drone ejaculates from one experimental apiary and concluded that most semen samples were contaminated. 

However, culture-based isolation techniques have limitations in species identification, as certain bacterial species are favored and others are difficult, including impossible, to culture in the laboratory. The advent of next-generation sequencing (NGS) has facilitated a more precise characterization of the microbiome, allowing for the analysis of large-scale amplicon sequencing and metagenomics research [5]. Metagenomics research has enhanced our knowledge of the microbiome, contributing to our understanding of the ecological dynamics within the host and the development of clinical and prophylactical treatments. Currently, only the gut microbiome of workers, queens and males has been characterized [7,8,9,10]. Nevertheless, to date, this technique has not been applied to the study of the semen microbiota in honey bee drones. 

This study was designed to characterize drone semen bacterial loads via a traditional culture technique and NGS and to describe its variation depending on the method of semen collection, the colony, apiary and year.

## 2. Materials and Methods

### 2.1. Animals and Semen Collection

The experiments were carried out during two beekeeping seasons (March–June 2022 and 2023) and included drones reared in honey bee (*Apis mellifera iberiensis*) colonies in northeastern Spain. In order to increase variability, an attempt was made to minimize the genetic relationships between the colonies used in this study using genealogical information. 

Mature flying drones were manually collected in the afternoon of days with good weather on their return to the hive, after blocking the entrance with a queen excluder. Subsequently, the drones were transported to the laboratory in polymethyl methacrylate-cages with absorbent paper at the bottom and a 96-well standard microplate filled with diluted honey [11] and were maintained at 32 °C until semen recovery, which was performed within the first 12 h after drone capture. 

### 2.2. Experiment 1: Assessing the Impact of Semen Collection Method on Bacterial Loads as Evaluated Using a Traditional Culture Technique

#### 2.2.1. Sample Collection

A total of 336 semen samples were obtained from drones originating from 8 colonies within the same apiary. Semen was extracted either from the seminal vesicles or from the ejaculates, and subsequently organized into pools comprising 8 drones each, resulting in a total of 42 pools, with 21 pools generated through each of the collection methods employed. This was accomplished over four sessions (5–6 replicas or pools per collection method each session). 

Ejaculation was induced within the first 24 h after drone collection using manual procedures [11]. An insemination syringe (Peter Schley, Lich, Germany) was used to collect semen in a capillary tube. For the collection of semen from the seminal vesicles, drones were decapitated, and their wings and legs were removed. Subsequently, the drones were affixed to a wax-filled Petri dish by securing them through the thorax with a needle. A dorsal window was then surgically created on the abdomen, allowing for the careful removal of the seminal vesicles after they were cut off at both ends using fine forceps. The intact seminal vesicles were then isolated and transferred to a microcentrifuge tube (0.5 mL) containing 200 µL of Kiev solution [12]. The semen from each seminal vesicle was extracted by gently squeezing it with the forceps, and the emptied seminal vesicles were subsequently discarded. All materials and media were sterilized before semen collection.

#### 2.2.2. Bacterial Culture and Sperm Quality Assessment

Each pool of semen samples was homogenized in 1 mL of brain heart infusion (BHI) broth (Scharlab, Barcelona, Spain) and vortexed for 1 min at the highest speed to ensure the suspension of adhering bacteria. Subsequently, decimal dilutions in phosphate-buffered saline (PBS) were plated on 13.5 cm diameter Petri dishes, each containing the following media: Plate Count Agar (BDTM) for aerobic mesophilic bacteria, MacConkey Agar (BDTM MacConkey II Agar) for Gram-negative enteric bacteria and Blood Agar (BDTM Columbia Agar with 5% Sheep Blood) for more fastidious microorganisms. The plates of MacConkey and Blood Agar were incubated at 37 °C and the Plate Count Agar plates at 30 °C, under aerobic conditions, for 48 h. The concentration (CFU/mL) of each type of bacteria was determined by plate counting and categorized into two groups for statistical analysis: <1000 CFU/mL and ≥1000 CFU/mL. The sperm quality assessment included the analysis of sperm motility, as described in [11,12], and sperm viability, as described in [11]. Briefly, for the sperm motility evaluation, semen was diluted in Kiev-BSA, placed in a prewarmed Makler^®^ chamber (MK; 10 µm deep; Sefi-Medical Instruments Ltd., Haifa, Israel) and live video pictures were recorded and analyzed using CASABee v2 software [11]. For sperm viability, semen was diluted in Kiev buffer, the spermatozoa were stained with a SYBR14-propidium iodide combination, photographed under the fluorescent microscope and examined using OpenCASA v2 software [13].

#### 2.2.3. Statistical Analysis 

The values obtained were expressed as mean ± standard deviation of the mean (SD). Statistical analyses were performed using the SPSS package, version 24.0 (IBM SPSS Statistics, Chicago, IL, USA). Normality distributions and the variance homogeneity of the median value score for each set were checked using the Kolmogorov–Smirnov and Levene tests, respectively. As all data were normally distributed, parametric tests were used throughout. Differences in membrane integrity and motility between groups were examined through a one-way analysis of variance (ANOVA) using generalized linear models. Statistical analyses were performed considering the parameters of sperm quality as the dependent variables and sample collection method and bacterial contamination with aerobic bacteria (<1000; ≥1000) as the independent factors. The statistical significance level (alpha) was set at 0.05.

### 2.3. Experiment 2: Study of the Microbiome of Ejaculated Drone Semen through the Use of an NGS Technique

#### 2.3.1. Sample Collection

Fifty semen samples were obtained through the process of drone ejaculation, with drones originating from 25 colonies (C1–C25) across five different apiaries (A1: Anies1, A2: Bolea, A3: Sabayes, A4: Anies2, A5: Nueno) over two successive years (2022 and 2023). The distance between the apiaries was between 1 km and 14 km. These semen samples were pooled to achieve a total volume of 8 µL, which aligns with the recommended volume for honey bee queen insemination [1]. Semen was diluted in 200 μL of Kiev buffer and was then frozen at −80 °C until DNA extraction for high-throughput sequencing. 

#### 2.3.2. Technical Approach

The composition and structure of the sampled microbial communities was assessed through the amplification and sequencing of the V3–V4 variable regions of the 16S rRNA gene, according to the previous methods described by Barba et al. [14].

Amplification was performed after 25 PCR cycles. In this procedure, positive and negative (Kiev buffer) controls were used to ensure quality control. The positive control is a Mock Community control (Zymobiomics Microbial Community DNA, 177 Catalog Nos. D6305, ZymoResearch, Irvine, CA, USA) and it was processed the same way as the samples. The obtained libraries were sequenced using Illumina Miseq (300 × 2) (Illumina Inc., San Diego, CA, USA).

#### 2.3.3. Bioinformatics Processing and Statistical Analysis

Raw demultiplexed forward and reverse reads were processed using QIIME2 (v2020.11) [15] and Dada2 software (plugin “dada2” from package “q2-dada2” v2020.11.1) [16].

The phylogenetical distances between OTUs were assessed using Mafft (v7.511) [17] and Fasttree 1.0 [18]. OTU and phylogenetic data were used to calculate the following beta diversity metrics: Unweighted UniFrac, Weighted UniFrac, Jaccard and Bray–Curtis.

Beta diversity distance matrices were used to perform a principal coordinates analysis (PCoA) and to make ordination plots using R software package version 4.2.0. The significance of the groups was tested using Permanova and ANOSIM tests. The Permdisp test was used to identify location vs. dispersion effects [19]. The significance threshold was set at 0.05.

The taxonomic assignment of OTUs was performed using a Bayesian Classifier [20] trained with Silva database version 138 (99% OTUs full-length sequences) [21].

The different statistical analyses were carried out using BiodiversityR version 2.14-1, PMCMRplus version 1.9.4, RVAideMemoire version 0.9-8 and vegan version 2.5-6 packages.

## 3. Results

### 3.1. Experiment 1

The collection method had a significant impact (*p* < 0.001) on the total number of aerobic mesophilic and blood agar bacteria in semen. Of the 42 semen samples analyzed, 26 (61.9%) were positive for aerobic mesophilic bacteria, which represented 100% of the samples obtained from the seminal vesicles (21 of 21) and 23.8% of those obtained from the ejaculates (5 of 21). The same number of contaminated samples were observed when using blood agar plates. In the contaminated samples, the degree of bacterial growth was much higher for the vesicular samples, with 2.23 × 10^6^ CFU/mL of aerobic mesophilic bacteria on average (range 0.2 to 8.2 × 10^6^), than for the ejaculates, with an average of 1736 CFU/mL aerobic mesophilic bacteria (range 40–3000). No bacterial growth was observed on the MacConkey agar plates (Gram-negative enteric bacteria) for the ejaculate samples, while 61.9% of the semen samples collected from the seminal vesicles were positive. 

The technique employed for semen collection and the level of the bacterial loads were not found to exert statistically significant effects on the evaluated sperm quality parameters (Table 1). 

### 3.2. Experiment 2

A total of 2,270,622 total reads were obtained before trimming. The number of reads obtained for the negative and Mock Community controls were as expected. The mock control was processed the same way as the samples. The profile obtained was consistent with the theoretical profile expected, validating the processes of library preparation. Bias is common and known between observed taxa in mock samples and theoretical expected mock compositions. Negative control samples were used to detect environmentally derived contaminants. Contaminant OTUs were defined as those that comprised less than two orders of magnitude difference in their abundance between negative controls and samples (mean value). These OTUs were removed from the dataset. After quality control, eight samples were removed (four from A2 (Bolea) and four from A4 (Anies2)), and 129,595 reads and 1494 OTUs were detected in the remaining 42 samples. Singletons and doubletons were also removed. Samples were subsampled up to 1177 reads to even the sample size and make quantitative comparisons. The minimum sequence length identified during subsampling was 301 bases.

#### 3.2.1. Diversity Analysis

**Figure 1:**
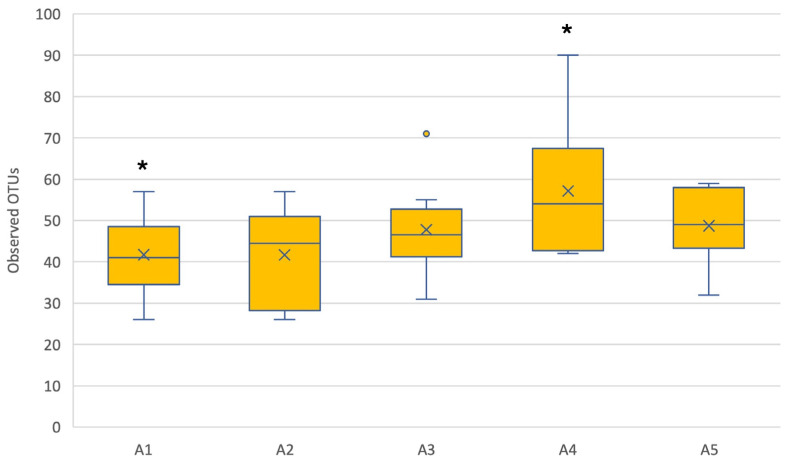
Observed Operational Taxonomic Units (OTUs) per apiary. Outliers are represented by yellow dots outside the whiskers. * indicates a significant differences between two apiaries (*p* < 0.05).

The richness per apiary fluctuated between 41.7 and 57.2 OTUs, as seen in Figure 1. Significant differences were observed in the richness of the microbiota between apiaries 1 and 4 (*p* < 0.05; Supplementary Table S1), while no differences in evenness were observed between apiaries (Supplementary Table S2), with an average Pielou index of 0.88. Significant differences (Supplementary Tables S3–S4) were also detected in the richness (Supplementary Figure S1) and evenness (Supplementary Figure S2) of the microbiota between hives. 

**Figure 2:**
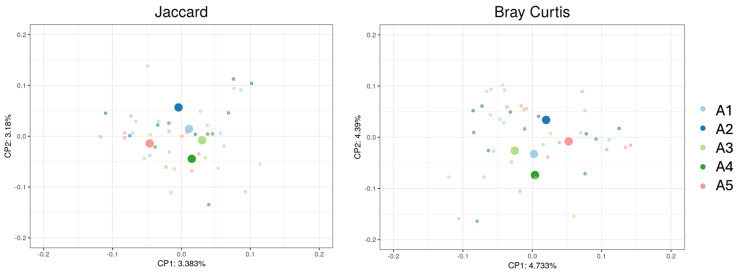
Principal coordinates analyses (PCoA) per apiary (A1, A2, A3, A4, A5) for Bray–Curtis and Jaccard distances. CP1 and CP2 represent the variation percentages of each axis. Each small dot represents one sample, while the bigger dots represent the centroids of each apiary. Dots ordinated closer to one another have microbial compositions more similar than those further away.

The apiary’s location had a significant effect on the community structure composition and abundance for Jaccard and Bray–Curtis distances but not for Unweighted and Weighted UniFrac. These were both dispersion and centroid differences, with a *p* < 0.05 for the Permanova test for Jaccard and Bray–Curtis (Supplementary Table S5) and *p* < 0.05 for Bray–Curtis and *p* < 0.001 for the Jaccard metrics for the Permdisp test (Supplementary Table S6). The ANOSIM test was also significant with an R close to 0 for these two metrics, indicating an even distribution of high and low ranks within and between groups (Supplementary Table S7). The PCoAs of the Bray–Curtis and Jaccard distances for each sample, classified by apiary, are represented in Figure 2.

#### 3.2.2. Taxonomic Profiles

**Figure 3:**
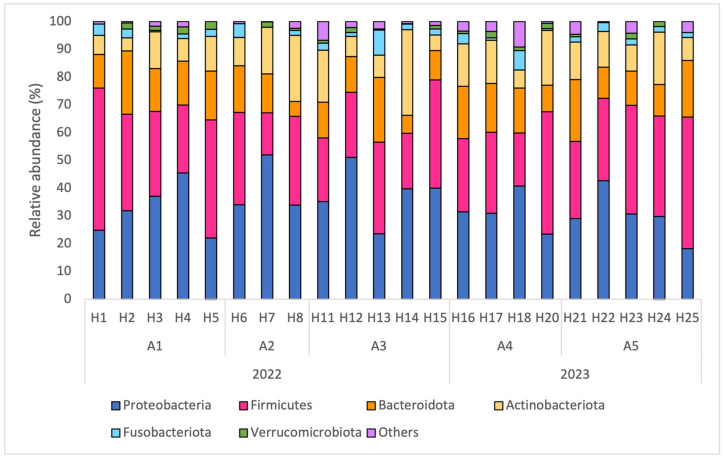
Relative abundance (RA) of the most abundant (RA > 1%) phyla, in % per hive (H1 to H25), in each apiary (A1 to A5) in the years 2022 and 2023.

The relative abundance (RA) of the most abundant phyla is shown in Figure 3. The most abundant bacterial phyla were Proteobacteria (34.0%), Firmicutes (31.9%), Bacteroidota (14.8%) and Actinobacteriota (13.0%). These four phyla represented more than 93% of the bacterial composition. Significant differences in phyla abundances were observed between colonies for the Bacteroidota and Firmicutes (Supplementary Table S8). No significant differences were observed between apiaries (Supplementary Table S9).

**Figure 4:**
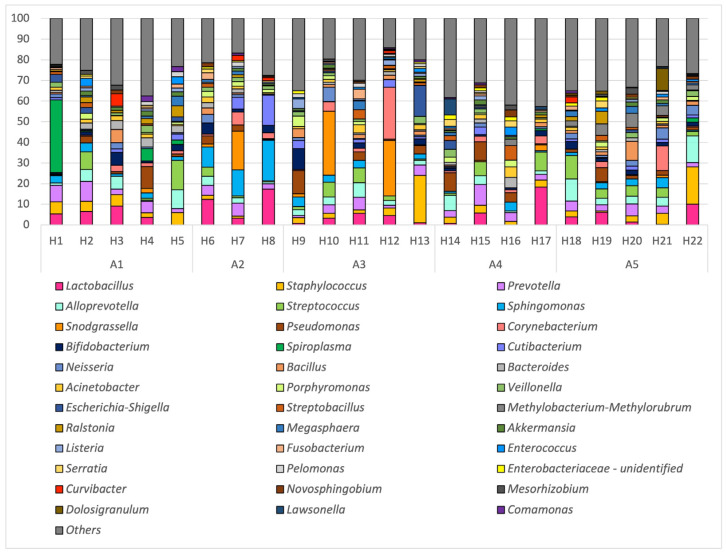
Relative abundance (RA) of the most abundant (RA > 0.5%) genera per hive (H1 to H25) in each apiary (A1 to A5) in year 2022 and 2023.

The RA of the most abundant genera is shown in Figure 4. The genera that represented less than 0.5% of the total were not included in the figure, although the sum of these genera still accounted for between 14.2% and 41.9% of the genera in each colony. Lactobacillus, Staphylococcus, Prevotella, Alloprevotella and Streptococcus were the most abundant genera. Lactobacillus encompassed 5.4% of the taxonomic composition, although this genus was absent in the semen collected from two colonies. Significant differences in genera abundances were observed between apiaries (Supplementary Table S10) and colonies (Supplementary Table S11) for the following genera: Sphingomonas (sixth most abundant genus), Methylobacterium-Methylorubrum (Beijerinckiaceae), Bifidobacterium (tenth most abundant genus) and Alloprevotella.

## 4. Discussion

The degree of the bacterial loads of semen may be influenced by several factors, such as the collection technique, as has been described in other species [22,23,24,25]. Two main techniques for collecting semen in honey bee drones have been described, based on the dissection of the seminal vesicles and the induction of ejaculation through the application of manual pressure to the thorax and abdomen [1]. The results obtained in this study clearly demonstrate that the collection method has a great impact on the degree of the bacterial loads of semen samples, with the traditional ejaculation method being more favorable than the collection of semen from the seminal vesicles. This is not surprising, as the collection of semen from the vesicles involves the opening of the abdominal cavity, which, even when performed with the utmost caution, as in the present study, carries a higher risk of contamination from both the external surface and the digestive tract of the drone.

In the current study, bacterial contamination was observed in 23.8% of the ejaculate pools when employing conventional culture methods. This finding contrasts with the previous work of Andere et al. [6], where the majority of samples (92.3%) showed bacterial contamination, despite the fact that a much smaller volume of semen (2 µL per pool vs. around 8 µL in the present study) was used. This discrepancy suggests that the seminal collection method employed may significantly influence the bacterial contamination of semen samples, thus emphasizing the crucial significance of maintaining optimal hygiene conditions throughout the process. Although the technique used for semen collection and the level of bacterial load did not exert statistically significant effects on the sperm quality parameters evaluated, it should not be ignored that obtaining semen from the dissection of the seminal vesicles could mean a greater risk of the transmission of bacterial pathogens to the queen.

Regarding the bacterial composition of the semen in honey bee drones, to the authors’ knowledge, this is the first study describing its microbiota. Previous metagenomic studies in honey bees have focused on the gut microbial communities [7,8,26,27,28,29]. It has been previously reported that adult honey bees show a core gut microbiota, regardless of external factors such as the environment and geographical location, composed of bacterial genera that include *Lactobacillus, Snodgrasella, Gilliamella, Bifidobacterium, Fructobacillus, Frischella, Bombella, Commensalibacter, Bartonella*, and *Parassachirabacter.* These bacterial species clusters seem to represent 95 to 99% of the gut microbiome [26,27]. In a study carried out in honey bee queens, it was seen that the most abundant taxa in the gut were *Acetobateraceae* (that includes genera such as *Bombella* and *Commensalibacter*) and *Lactobacillaceae*, although the latter reduced its abundance with age [28]. Honey bee queens seem to lack the stable core microbiota associated with workers and show a microbiota lacking diversity and consistency compared to worker bees of similar ages [28,29]. Nevertheless, it has been seen that the gut microbiota of drones and queens contain most of the same bacteria as workers, although important differences were observed between males and workers when the influence of the most abundant OTUs was reduced. Honey bee workers also seem to display higher microbial diversity compared to the drones’ hindgut microbiota [8].

In our study, *Snodgrasella* was an important genus, with an RA = 4%, *Bifidobacterium* as well (RA = 2.5%), while the most abundant was *Lactobacillus,* with an RA = 5.4%. *Bombella* was only present in one individual with a low RA, while *Commensalibacter* was present in six male individuals with a low RA too. *Frischella* was present in two drones. No other abundant genera were shared between the honey bee gut microbiota and the semen microbiota, as *Gilliamella* and *Fructobacillus* were not identified in drone semen. 

The discovery of *Listeria* spp. (RA = 0.9%) in drone semen is interesting as it is the first description of this genus in honey bees. This is a genus that includes pathogenic and non-pathogenic species [30] that are often described in the feces and gastrointestinal tract of mammals [31]. Species such as *Listeria marthii*, one of two species isolated in one specimen in our study, were previously isolated in soil, stagnant water and running water samples obtained from the natural environment in only one study [32]. The other *Listeria* sp. present in several individuals in the present study remained unidentified. It would not be strange for bees to ingest this type of bacterial genus into their microbiota through the ingestion of environmental water. Our results suggest the honey bee as a potential host that should be taken into account in the ecology of *Listeria* spp.

The microbial community structure was significantly affected by the apiary location, based on the results of our beta diversity metrics (Jaccard and Bray–Curtis), indicating a different species composition between apiaries. Indeed, several colonies had significant differences in the abundance of important genera such as *Sphingomonas*, *Bifidobacterium* and *Alloprevotella*. This could suggest that geographical location does influence the microbiota of the semen in drones. It has been previously reported that workers of the same age within a colony can display different abundances of the bacteria species present in their core gut microbiota [27].

When compared with the semen microbiota of other species, such as the human species, *Lactobacillus*, *Staphylococcus*, *Prevotella*, *Streptococcus* and *Corynebacterium* are amongst the ten most abundant genera in our study and in male semen [33,34,35]. Other abundant genera identified in our study (Figure 4) and in male semen include *Ralstonia* [34], *Enterococcus* [33], *Veillonella* [33,34], *Fusobacterium* [33], *Porphyromonas* [34], *Novosphingobium* [34], *Pseudomonas* [34,35], *Bacillus* and *Escherichia* [35]. Three studies reported clusters in the semen microbiome of men. One cluster was dominated by *Lactobacillus,* while the other by *Prevotella* [33,34,35]. *Prevotella* was associated with low-quality sperm and *Lactobacillus* was associated with the normal parameters of sperm quality [33,35].

Some bacterial species present in drone semen could be linked to the antimicrobial activity of seminal fluid against the sexually transmitted parasite *Nosema apis*, which has been related to the secretion of certain proteins such as chitinase [36]. This protein is produced by *Serratia* and *Bacillus*, and both genera had an overall RA > 0.5% in drone semen (Figure 4). The high abundance of *Lactobacillus* in drone semen should also be taken into account due to the known antimicrobial potential that lactic acid bacteria can display through various mechanisms [37]. As previously mentioned, they have been linked to normal semen morphology and quality in the human species and they have also been linked to the ability to restrict sperm lipid peroxidation and therefore significantly maintain sperm motility and viability under induced oxidative conditions in vitro [38].

## 5. Conclusions

In conclusion, the collection method used has a great impact on the degree of the bacterial loads in semen samples, with the traditional ejaculation method being more favorable than the collection of semen from the seminal vesicles. There are differences between apiaries and colonies in the composition and abundance of the seminal microbiota. Despite this, more research is needed to explore the potential use of seminal microbiota members as probiotics in honey bees, and this study represents an important step forward in the area. Further studies should also focus on the association of certain bacterial clusters with abnormal sperm quality in drones. 

## Figures and Tables

**Table 1:** Sperm quality parameters (mean ± SD) in honey bee drone semen depending on the semen collection method and bacterial loads.

Sample Characteristic	Sperm Motility (%)	Sperm Viability (%)
Seminal vesicle ^1^	78.50 ± 4.75%	72.20 ± 5.79%
Ejaculate ^2^	80.33 ± 5.51%	74.02 ± 6.99%
Slightly contaminated ^3^	80.07 ± 5.91%	74.19 ± 7.22%
Highly contaminated ^4^	78.93 ± 4.61%	72.31 ± 4.61%

^1^ Semen collected from the seminal vesicles. ^2^ Semen collected from the ejaculates. ^3^ Low-bacterial-contamination semen samples (<1000 CFU/mL). ^4^ High-bacterial-contamination semen samples (≥ 1000 CFU/mL). No significant differences in sperm quality variables were observed between the different collection methods and bacterial loads.

## Data Availability

The authors confirm that the data supporting the findings of this study are available upon reasonable request from the corresponding author.

## Supplementary Materials

The following supporting information can be downloaded at https://www.mdpi.com/article/10.3390/insects15060377/s1, Figure S1: observed Operational Taxonomic Units (OTUs) per hive (H); Figure S2: evenness (Pielou index) per hive (H), values range from 0 (uneven) to 1 (even); Table S1: richness comparisons between apiaries; Table S2: evenness comparisons between apiaries; Table S3: richness comparisons between hives; Table S4: evenness comparisons between apiaries; Table S5: Permanova test of dissimilarity indices; Table S6: Permdisp test of dissimilarity indices: Table S7: ANOSIM test of dissimilarity indices; Table S8: phylum comparisons between hives; Table S9: phylum comparisons between apiaries; Table S10: genus comparisons between apiaries; Table S11: genus comparisons between hives.
