# Supplementary figures and images for: Microbiota Analysis of Ejaculated Honey Bee Drone Semen and the Effect of Semen Collection Method on Bacterial Loads
Source: Insects. 2024 May 22;15(6):377. doi: 10.3390/insects15060377 (PMC11203891; doi:10.3390/insects15060377)

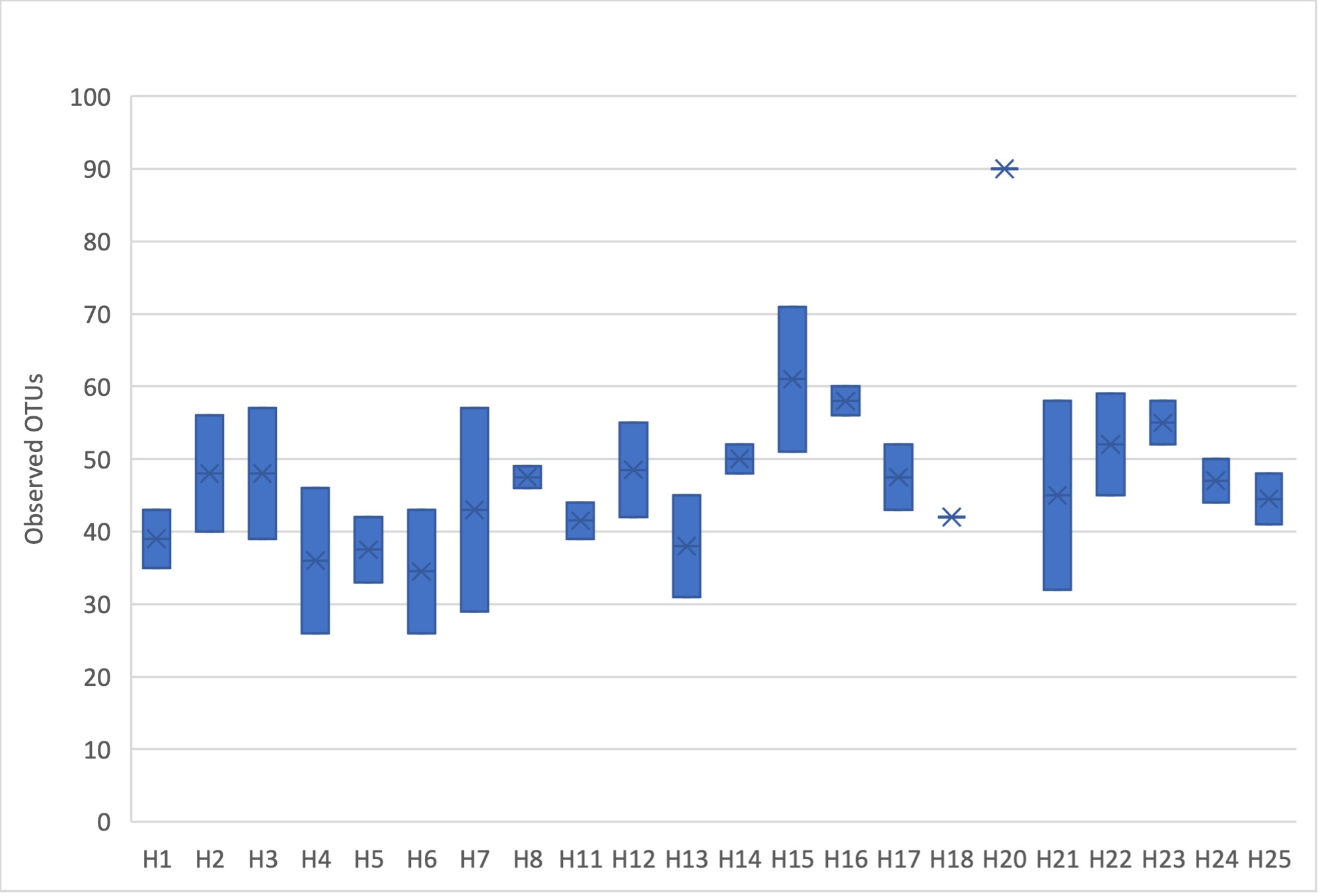

Supplement: Supplementary file 1 [file insects-15-00377-s001.zip › Supplementary Figure 1.jpg]

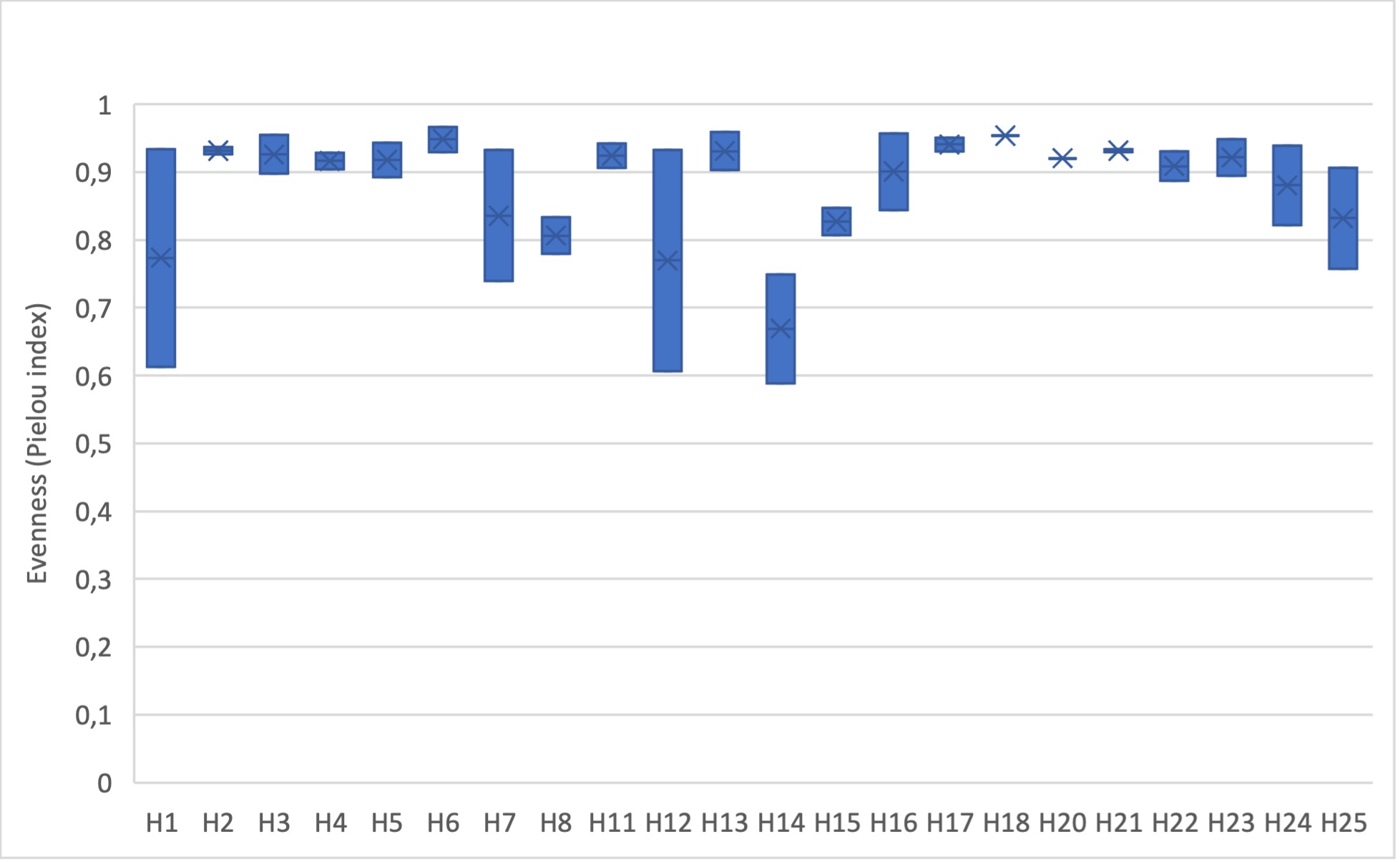

Supplement: Supplementary file 1 [file insects-15-00377-s001.zip › Supplementary Figure 2.jpg]
